# A randomized, double-blind study of AMG 108 (a fully human monoclonal antibody to IL-1R1) in patients with osteoarthritis of the knee

**DOI:** 10.1186/ar3430

**Published:** 2011-07-29

**Authors:** Stanley B Cohen, Susanna Proudman, Alan J Kivitz, Francis X Burch, John P Donohue, Deborah Burstein, Yu-Nien Sun, Christopher Banfield, Michael S Vincent, Liyun Ni, Debra J Zack

**Affiliations:** 1 Rheumatology, Metroplex Clinical Research Center, 8144 Walnut Hill Lane, Dallas, TX 75231, USA; 2 FRACP, Bone Densitometry Research, Royal Adelaide Hospital, North Terrace, Adelaide SA 5000, Australia; 3 Arthritis and Osteoporosis, Altoona Center for Clinical Research, 175 Meadowbrook Lane, Duncansville, PA 16635-8445, USA; 4 San Antonio Center for Clinical Research, 7940 Floyd Curl Drive, San Antonio, TX 78229, USA; 5 Rheumatologic and Immunologic Disease, Cleveland Clinic, 2950 Cleveland Clinic Boulevard, Florida, Weston, FL 33331, USA; 6 Radiology, Beth Israel Deaconess Medical Center, 330 Brookline Drive, Boston, MA 02115, USA; 7 Inflammation Research, Amgen, Inc., One Amgen Center Drive, Thousand Oaks, CA 91320, USA; 8 Medical Sciences Early Development, Amgen, Inc., One Amgen Center Drive, Thousand Oaks, CA 91320, USA; 9 Global Development, Amgen, Inc., One Amgen Center Drive, Thousand Oaks, CA 91320, USA; 10 Global Biostatistical Science, Amgen, Inc., One Amgen Center Drive, Thousand Oaks, CA 91320, USA

## Abstract

**Introduction:**

AMG 108 is a fully human, immunoglobulin subclass G2 (IgG2) monoclonal antibody that binds the human interleukin-1 (IL-1) receptor type 1, inhibiting the activity of IL-1a and IL-1b. In preclinical studies, IL-1 inhibition was shown to be beneficial in models of osteoarthritis (OA). The purpose of this two-part study was to evaluate the safety and pharmacokinetics (PK; Part A) and clinical effect (Part B) of AMG 108 in a double-blind, placebo-controlled, multiple-dose study in patients with OA of the knee.

**Methods:**

In Part A, patients received placebo or AMG 108 subcutaneously (SC; 75 mg or 300 mg) or intravenously (IV; 100 mg or 300 mg) once every 4 weeks for 12 weeks; in Part B, patients received placebo or 300 mg AMG 108 SC, once every 4 weeks for 12 weeks. The clinical effect of AMG 108 was measured in Part B by using the Western Ontario and McMaster Universities (WOMAC) osteoarthritis index pain score.

**Results:**

In Part A, 68 patients were randomized, and 64 received investigational product. In Part B, 160 patients were randomized, and 159 received investigational product. AMG 108 was well tolerated. Most adverse events (AEs), infectious AEs, serious AEs and infections, as well as withdrawals from the study due to AEs occurred at similar rates in both active and placebo groups. One death was reported in an 80-year-old patient (Part A, 300 mg IV AMG 108; due to complications of lobar pneumonia). AMG 108 serum concentration-time profiles exhibited nonlinear PK. The AMG 108 group in Part B had statistically insignificant but numerically greater improvement in pain compared with the placebo group, as shown by the WOMAC pain scores (median change, -63.0 versus -37.0, respectively).

**Conclusions:**

The safety profile of AMG 108 SC and IV was comparable with placebo in patients with OA of the knee. Patients who received AMG 108 showed statistically insignificant but numerically greater improvements in pain; however, minimal, if any, clinical benefit was observed.

**Trial Registration:**

This study is registered with ClinicalTrials.gov with the identifier NCT00110942.

## Introduction

Osteoarthritis (OA) is a chronic, painful, and potentially disabling disease of the joints that is manifested by cartilage damage, changes in the underlying bone, and varying degrees of synovial inflammation. The prevalence of OA increases with age; 60% to 70% of individuals aged 70 to 80 years have pathologic evidence of OA [1].

The exact cause of OA is unknown. Recent debate suggests that cytokines produced by activated synovial cells or articular cartilage may be as important in the pathogenesis of OA as a concomitant response to mechanical forces or molecular events from the cartilage and synovium [2]. Cytokines such as interleukin-1 (IL-1) stimulate the synthesis of proteolytic enzymes such as matrix metallo-proteinases, nitric oxide (NO), prostaglandins, and other mediators and effectors of tissue destruction [3]. IL-1 also inhibits chondrocyte repair of degraded cartilage extracellular matrix [4]. In animal models, IL-1 has been shown to induce cartilage damage, as measured by glycosaminoglycan (GAG) release, in a NO-dependent manner [5,6]. A relative deficiency of endogenous IL-1 receptor antagonist (IL-1ra), the natural antagonist to IL-1 beta (IL-1β), has been found in the synovial fluid [7] and diseased cartilage tissue of patients with OA [8]. Cartilage from OA patients who had undergone joint-replacement surgery has also been shown to respond to IL-1β stimulation with higher NO production than RA cartilage [8]. Animal studies have suggested that intraarticular (IA) injections of IL-1ra may slow the progression of cartilage lesions in OA [9-12]. These findings suggest that blocking the activity of IL-1β may protect against structural changes in OA [13,14]. Finally, IL-1 antagonists may also play a role in the pain of OA [15]. In a small study of patients with OA, IA injections of the competitive inhibitor of IL-1, anakinra, were well tolerated and contributed to some improvements in their pain [16].

AMG 108 is a fully human, immunoglobulin subclass G2 (IgG2) monoclonal antibody that binds the third immunoglobulin domain of the interleukin-1 receptor type 1 (IL-1R1) and nonselectively inhibits the activity of both forms of IL-1 (IL-1α and IL-1β). Inhibiting the proinflammatory effects of these IL-1 isoforms with AMG 108 may be useful in treating OA.

The objectives of this two-part study were to compare the safety and pharmacokinetics (PK) of AMG 108, given either subcutaneously (SC) or intravenously (IV), in a multiple-dose, dose-ranging study (Part A), and to determine the clinical effect (by using the Western Ontario and McMaster Universities (WOMAC) osteoarthritis index pain score) of multiple administrations of a selected dose of AMG 108 versus placebo given SC to patients with active OA of the knee (Part B).

## Materials and methods

### Patients

Eligible patients were 30 years old or older and had OA of the knee that met the 1987 American College of Rheumatology (ACR) [17] classification criteria (knee pain, radiographic osteophytes, and one or more of the following: age older than 50 years; morning stiffness, 30 minutes or less; crepitus on motion); and radiographic evidence of tibiofemoral-compartment knee OA within 12 weeks of screening. An index knee was identified at baseline for all study evaluations of clinical benefit; in addition to this diagnosis of OA, patients in Part A were required to have the presence of a knee effusion in the index joint, and patients in Part B were required to have index knee pain at a level more than 30 mm on 100-mm visual analog scale (VAS).

Patients who had been taking any over-the-counter nutritional supplements, or nonprescribed supplements (for example, glucosamine, chondroitin sulfate, shark cartilage, diacerhein, soya extract), or nonsteroidal anti-inflammatory drugs (NSAIDs) must have been taking a stable dose for > 2 months; any utilization of physical therapy, biomechanical devices, or orthotic support also must have been stable for > 2 months. Patients who were receiving NSAID therapy must have discontinued therapy for at least 5 half-lives of the particular NSAID before randomization into the study.

Patients were excluded if their weight was > 125 kg or if they had end-stage or bone-on-bone OA (Kellgren-Lawrence 4), symptomatic hip OA ipsilateral to the index knee, isolated OA of the femoropatellar joint, inflammatory arthropathy, or diagnosis of a condition other than knee OA that the investigator thought could cause or affect pain in the index knee. Patients also were excluded if they had received any previous AMG 108, anakinra, or other experimental IL-1 inhibitor; or, at the time of study entry, had received an investigational monoclonal antibody within 6 months; had received viscosupplementation therapy within 3 months; had participated in a trial of an investigational drug or device within 2 months, had received an IA or systemic corticosteroid injection within 1 month; or were using neuromodulatory agents as analgesic therapy for OA. They could not have had a malignancy within 5 years (with the exception of basal cell or *in situ *cancer); history of recurrent chronic infections, active tuberculosis, or antibodies to human immunodeficiency virus or hepatitis C; known or suspected susceptibility to infectious disease; significant hematologic disease; elevated serum creatinine or liver-function tests (≥1.5 times upper limit of normal); uncontrolled or clinically significant systemic disease (for example, diabetes mellitus, cardiovascular disease, or hypertension); or any other condition that, in the opinion of the investigator, would interfere with the interpretation of the study results. Women were excluded if they were pregnant or nursing or were not using adequate contraception (if of childbearing potential).

Particular attention was given to patient neutrophil counts, with enrollment into the study to be stopped by the data-monitoring committee if an increased rate of neutropenia was observed. Specifically, for each patient, neutrophil counts were analyzed 1 to 2 days before dosing (days 28 and 56); and dosing was stopped if the predose neutrophil count was < 1.00 × 10^9^/L.

### Study design

This was a two-part, randomized, double-blind, placebo-controlled, multiple-dose study in patients with OA. In Part A, the dose-ranging portion of the study, 64 patients were randomized 3:1 in each of four cohorts (12 active; four placebo) to receive AMG 108 SC (75 mg or 300 mg) or IV (100 mg or 300 mg) or placebo every 4 weeks for 12 weeks (for a total of three doses of investigational product). In Part B, 160 patients were randomized 1:1 to receive 300 mg AMG 108 SC or placebo, by using the same dosing schedule.

For the 10- and 300-mg IV dose groups, AMG 108 (30 mg/ml) or placebo in sterile 5% dextrose in water (D5W) was administered as a 100-ml IV infusion over a 30-minute period by using a peristaltic pump. For the 75-mg and 150-mg SC dose groups, AMG 108 (100 mg/ml) or placebo was administered as a single, SC injection (0.75 ml or 1.5 ml, respectively) in the subject's anterior abdominal wall. For the 300-mg SC dose group, AMG 108 (100 mg/ml) or placebo was administered as two 1.5-ml SC injections at approximately the same time of day and at least 2 cm apart on the anterior abdominal wall.

Efficacy analyses in Part B included two substudies: a main substudy using the WOMAC osteoarthritis index pain score conducted at all study sites (145 patients), and a minor substudy of the delayed gadolinium-enhanced magnetic resonance imaging of cartilage (dGEMRIC) conducted at only one study site (because only 15 of the desired 30 patients were enrolled, the sample was too small to draw meaningful conclusions; however, for completeness, dGEMRIC methods and results are included in Additional File 1). After administration of the last dose in both Part A and Part B, all patients were followed up for 8 weeks ( < 300-mg dose cohorts) or 12 weeks (300-mg dose cohorts).

The study was conducted according to the Declaration of Helsinki and the International Conference on Harmonisation Tripartite Guideline on Good Clinical Practice. Approvals from appropriate research ethics committees were obtained from each participating study center. All patients provided written informed consent before participating. An external Data Monitoring Committee monitored patient safety throughout the study.

### End points

The primary end point in Part A of the study was the safety of AMG 108; additional end points included the PK) of AMG 108. In Part B, the primary end point was the clinical efficacy of AMG 108 (change from baseline to week 6 in the WOMAC pain score); additional end points included the safety and PK of AMG 108.

Safety end points in both parts of the study included treatment-emergent adverse events (AEs), infectious AEs, serious AEs and infections, injection-site reactions, and laboratory abnormalities.

In Part A of the study, serum samples were collected from all patients for analysis of AMG 108 concentrations at the following predefined time points: predose on day 1; and postdose day 1 at 30 minutes and 8 hours, and on days 2, 3, 7, 14, 21, 28, and 41; predose on day 56; and postdose day 56 at 30 minutes and 8 hours, and on days 57, 58, 63, 70, 77, 84, 98, and 112. Patients receiving > 100-mg doses also had serum collected on days 126 and 140. A subset of patients in the first two cohorts had additional samples collected at prespecified time points more frequently during the 2 days after the first dose (day 1) and the third dose (day 56). Synovial fluid samples were collected at screening for all patients and at one of the following time points (*n *= 4 per time point): day 2, 7, 14, or 28. In Part B of the study, serum samples were collected for PK analysis at day 1 (predose), and at weeks 2, 4, 6, 8, 10, 12, 14, 16, and 20. Synovial fluid samples were not collected in Part B.

In Part B, the primary efficacy end point was the change from baseline to week 6 in the WOMAC pain score. Other efficacy end points included change in subcomponent (function and stiffness) and composite WOMAC scores from baseline to weeks 6 and 12. Change in physician and patient global assessments of pain, the SF-36, and the EQ-5D were also assessed in Part B.

### Biochemical analysis

Serum samples were analyzed for AMG 108 concentrations by using a validated enzyme-linked immunosorbent assay (ELISA) at MDS Pharma Services (Quebec, Canada). Non-compartmental analyses were performed by using WinNonlin Professional Software (Version 4.1e, Pharsight Corporation, Mountain View, CA) to estimate the maximum observed serum concentration after dosing (C_max_), the time to reach C_max _(t_max_), the observed serum concentrations of AMG 108, the area under the concentration-time curve during the dosing interval (AUC_0-τ_), and the trough concentration after the first and third doses.

C-reactive protein (CRP) was determined by using a highly sensitive latex particle-enhanced immunoturbidimetric assay that compares the laboratory sample with standard CRP dilutions. The assay was performed at ICON Clinical Research (Farmingdale, NY). Elevated bilirubin, hemoglobin, and lipids did not interfere in the assay.

### Statistical analysis

Patient data were analyzed according to randomized treatment arm regardless of actual treatment received during the study. The safety dataset included all patients who received ≥1 dose of investigational product.

Efficacy end points in Part A were exploratory end points, and sample size in Part A was too small for inferential statistical analysis on the efficacy end points. In Part B, the data set used for analysis of the primary efficacy end point included all Part B randomized patients who participated in the WOMAC study (*n *= 145). The primary end point was the change in the WOMAC pain score from day 1 to week 6 in patients administered AMG 108 compared with placebo. Assuming an effect size of 0.60 (that is, the expected difference in means between the placebo arm and AMG 108 arm is 60 on a 500-point WOMAC), a sample size of 66 patients per arm (300 mg and placebo) would have ≥90% power to detect, at the 5% significance level, a difference between the AMG 108 arm and the placebo arm by using a Wilcoxon rank-sum test. The total sample size was adjusted for a possible 10% drop-out rate during the study. For the primary analyses of efficacy in Part B of the study, the last-observation-carried-forward (LOCF) method was used to impute missing data; observed data also were presented. The data set used for analysis of the secondary and non-MRI related exploratory efficacy end points included all randomized patients (*n *= 160). Stratification of patients by baseline WOMAC was performed as a *post hoc *analysis.

## Results

### Patient disposition and disease characteristics

**Figure 1:**
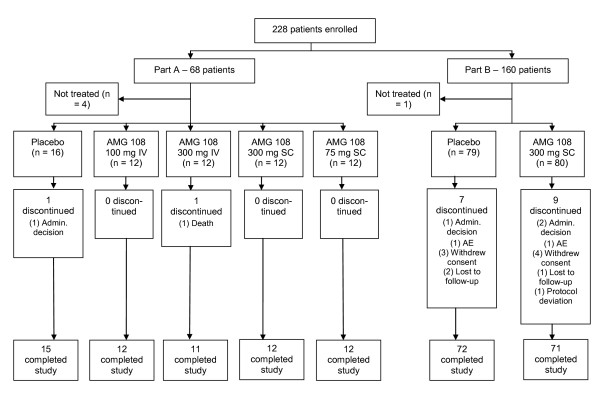
**CONSORT diagram**. Admin. Decision, administrative decision; AE, adverse event; IV, intravenous; SC, subcutaneous.

Patient disposition is presented in Figure 1. In Part A, 68 patients were randomized, four patients did not receive investigational product because of ineligibility, and 64 patients were dosed. Two patients did not complete Part A of the study: one died on day 53 after receiving two doses of 300 mg AMG 108 IV; the other discontinued because of personal reasons. In Part B, 80 patients were randomized to 300 mg AMG 108 SC, and 80 patients, to placebo; of the 160 patients, 145 were randomized to the WOMAC substudy, and 15 were randomized to the dGEMRIC study. Study completion in Part B was similar between the treatment groups: 159 patients (99%) received one or more doses of investigational product; 88.8% of patients in the AMG 108 group, and 90.0% in the placebo group completed the study.

Demographics and baseline disease characteristics were well balanced among the groups (Table 1). The majority of patients were women (Part A, 66%; Part B, 68%), and most were white (Part A, 98%; Part B, 83%). The mean age was 61 years for patients in both parts of the study. The mean duration of OA at baseline of Part A was 8 years for the AMG 108 group and 10 years for the placebo group; for Part B, it was 6 years for both groups.

**Table 1:** Baseline demographics and disease characteristics

	Part A						Part B	
		AMG 108						
	Placebo (*n *= 16)	100 mg IV (*n *= 12)	300 mg IV (*n *= 12)	300 mg SC (*n *= 12)	75 mg SC (*n *= 12)	All AMG 108 (*n *= 48)	Placebo (*n *= 80)	AMG 108 300 mg SC (*n *= 80)
Mean age (years)	60.8	61.1	62.8	59.6	62.3	61.4	60.1	61.3
Female, *n *(%)	10 (63)	11 (92)	7 (58)	5 (42)	9 (75)	32 (67)	54 (68)	54 (68)
Ethnicity, *n *(%)								
White	15 (94)	12 (100)	12 (100)	12 (100)	12 (100)	48 (100)	66 (83)	67 (84)
Black	1 (6)	0	0	0	0	0	2 (3)	7 (9)
Hispanic	-	-	-	-	-	-	12 (15)	6 (8)
Mean weight (kg)	83.8	79.9	90.7	85.5	82.4	84.6	87.6	88.2
Mean BMI (kg/m^2^)	30.4	30.8	31.9	29.8	30.9	30.8	31.9	32.0
Duration of OA (years)	9.6	6.9	10.2	6.6	10.0	8.4	6.1	6.1
Kellgren-Lawrence score (*n *(%))								
1	2 (13)	3 (25)	1 (8)	0	3 (25)	7 (15)	4 (5)	1 (1)
2	4 (25)	3 (25)	7 (58)	5 (42)	4 (33)	19 (40)	30 (38)	40 (50)
3	10 (63)	6 (50)	4 (33)	7 (58)	5 (42)	22 (46)	46 (58)	39 (49)

BMI, body mass index; IV, intravenous; OA, osteoarthritis; SC, subcutaneous.

No patients in Part A of the study were taking NSAIDs at baseline; however, the majority (63%) of patients in Part B were taking NSAIDs before enrollment in the study. Patients were required to discontinue NSAIDs within at least 5 half-lives before randomization; NSAIDs and or analgesics were not allowed except as rescue therapy during the study.

### Safety

AMG 108 was well tolerated during the two-part study. Most AEs, infectious AEs, serious AEs and infections, and withdrawals from study due to AEs occurred at similar rates in the AMG 108 and placebo groups (Table 2).

**Table 2:** Summary of adverse events

	IV Administration				SC Administration		
Number (%) of patients with	Placebo (*n *= 8)	100 mg (*n *= 12)	300 mg (*n *= 12)	All AMG 108 (*n *= 24)	Placebo (*n *= 88)	75 mg (*n *= 12)	300 mg (*n *= 94)
Any adverse event	8 (100)	12 (100)	12 (100)	24 (100)	74 (84)	12 (100)	77 (82)
Most common AE							
Headache	4 (50)	9 (75)	7 (58)	16 (57)	22 (25)	4 (33)	15 (16)
Upper respiratory tract infection	1 (13)	2 (17)	5 (42)	7 (29)	7 (8)	6 (50)	9 (10)
Infection	3 (38)	4 (33)	8 (67)	12 (50)	18 (21)	7 (58)	25 (27)
Injection-site reaction	1 (13)	1 (8)	2 (17)	3 (13)	3 (3)	0	7 (7)
Treatment-related AE	5 (63)	8 (67)	6 (50)	14 (58)	10 (11)	0	28 (30)
AE leading to study discontinuation	0	0	0	0	1 (1)	0	1 (1)
Serious AE	0	0	2 (17)	2 (8)	3 (3)	0	2 (2)
Treatment-related SAE	0	0	1 (8)	1 (4)	0	0	1 (1)
Serious infectious AE	0	0	1 (8)	1 (4)	2 (2)	0	1 (1)
Death	0	0	1 (8)	1 (4)	0	0	0

AE, adverse event; IV, intravenous; SAE, serious adverse event; SC, subcutaneous. SAEs were reported by two patients in the 300-mg IV group (hemorrhagic diarrhea and unstable angina in one; and lobar pneumonia, respiratory failure, multiorgan failure, sepsis, neutropenia, and leukopenia in the other); two patients in the 300-mg SC group (pancreatitis in one; and pneumonia and supraventricular tachycardia in the other); and three patients in the placebo SC group (arthropod bite and Staphylococcus infection in one; abdominal pain in the second; and coronary artery disease in the third). All serious infectious AEs have been listed as SAEs above and include one patient in the 300-mg IV group (lobar pneumonia and sepsis); one patient in the 300-mg SC group (pneumonia); and two patients in the placebo SC group (Staphylococcus infection in one and abdominal pain in the other).

One patient in Part A (AMG 108, 300 mg IV), an 80-year-old man with an ongoing history of hypertension and asthma at study entry, died during the study. He had mild neutropenia (absolute neutrophil count (ANC) 1.92 × 10^9^/L) and symptoms of upper respiratory infection before his second dose of study drug. One week after a second dose of AMG 108, the patient was hospitalized with respiratory failure due to lobar pneumonia; at the time of admission, his ANC was 1.3 × 10^9^/L. By the following day, the ANC had decreased to 0.4 × 10^9^/L; two days later, his ANC increased to 8.9 × 10^9^/L and further rebounded to 24.3 × 10^9^/L. A bacterial agent was not identified. Despite intensive resuscitative and life-support treatment, the patient's condition worsened, and he died, 25 days after the last injection of AMG 108, of lobar pneumonia, respiratory failure, multiorgan failure, and sepsis, which the investigator considered related to the investigational product.

Total numbers of adverse events between 300 mg SC AMG 108 administration and SC placebo administration (the two largest groups) were well balanced at 82% (77 of 94) and 84% (74 of 88), respectively. The overall incidence of infections in these two SC groups appeared to be higher in the AMG 108 group at 27% (25 of 94) versus 21% (18 of 88) for placebo; however, the incidence of serious infectious AEs was 1% (1 of 94) for AMG 108 and 2% (2 of 88) for placebo. The most frequently reported infectious AE was upper respiratory infection (URI) in 10% (9 of 94) of the 300-mg SC AMG 108 group and 8% (7 of 88) of the placebo SC group. One death occurred in the entire study, as described earlier.

Injection-site reactions also occurred more frequently in the AMG 108 group than in the placebo group (7% versus 3%, respectively, in Part B; Table 2), but most were mild or moderate in severity, and no patient withdrew from the study because of an injection-site reaction.

**Figure 2:**
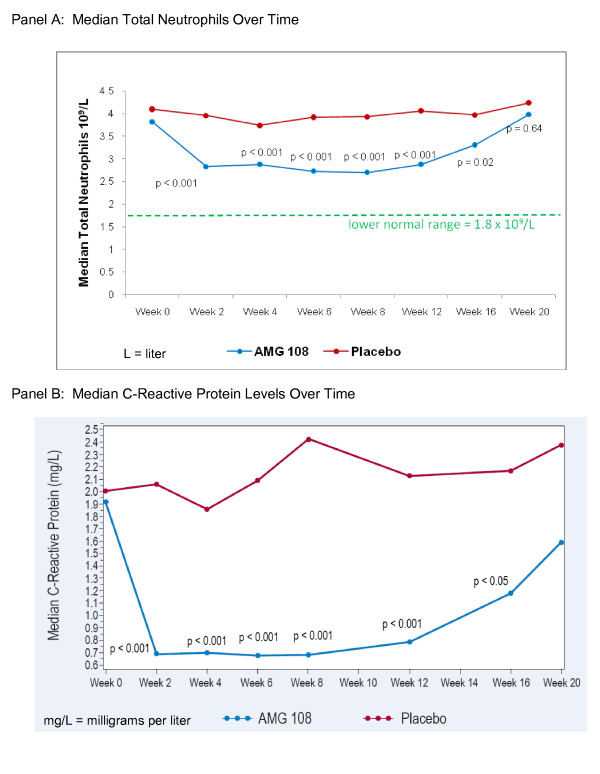
**Laboratory values over time, Part B**. **(a) **Absolute neutrophil count (ANC); **(b) **C-reactive protein levels over time.

No clinically significant changes in clinical laboratory results were observed, with the exception of expected decreases in ANC in the AMG 108 groups of both Part A and Part B of the study. In Part B, mean neutrophil counts at baseline were 4.17 and 4.29 × 10^9^/L for AMG 108 and placebo cohorts, respectively. At week 6, the mean neutrophil counts had decreased to 2.95 × 10^9^/L for the AMG 108 cohorts, but were only slightly lower than at baseline in the placebo group (4.03 × 10^9^/L). By week 20 (end of study), the neutrophil counts had essentially returned to baseline: 4.02 × 10^9^/L for the AMG 108 cohorts, and 4.10 × 10^9^/L for the placebo cohort. In all of Part B, only two patients had a reversible decrease in ANC below 1.0 × 10^9^/L (but above 0.5 × 10^9^/L) at the lowest measurement, and 11 patients had a reversible ANC decrease between 1.5 and 1.0 × 10^9^/L. Neutrophil counts of all patients returned to baseline levels as early as 2 weeks but within 8 weeks after the last injection of AMG 108 (Figure 2a).

Because suppression of CRP levels is known to be a pharmacodynamic effect of IL-1 inhibition, a highly sensitive CRP assay was used as part of the chemistry evaluations. In Part B patients, a significant (*P *< 0.001) difference in median CRP levels favoring AMG 108 over placebo was observed from week 2 to week 12 and was maintained at week 16 (*P *< 0.05), 8 weeks after the last injection (Figure 2b).

### Pharmacokinetics

**Figure 3:**
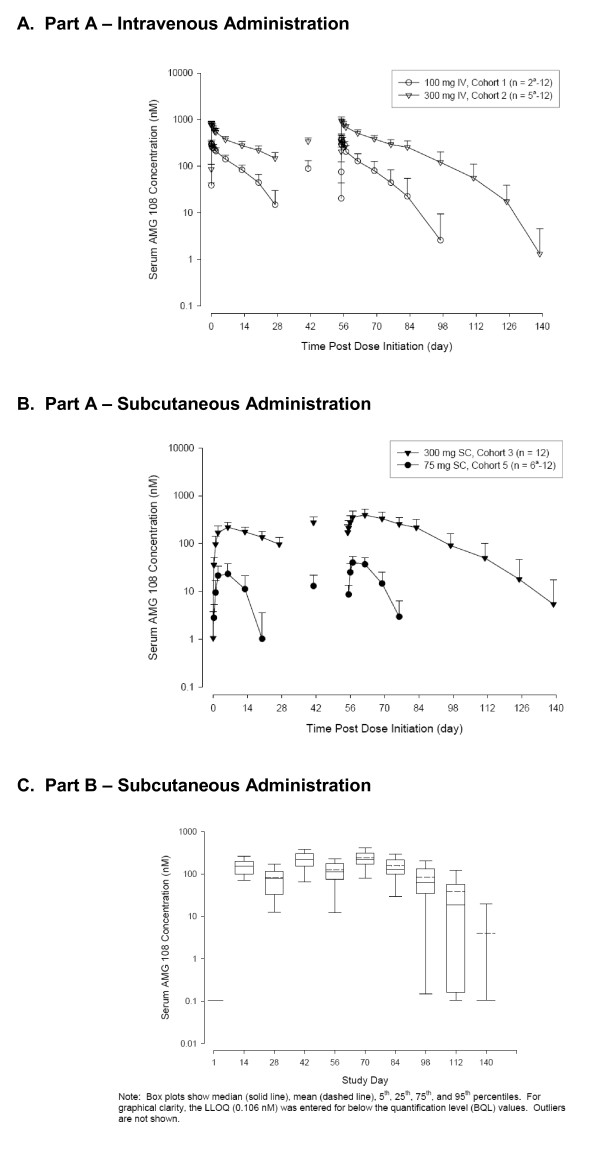
**Pharmacokinetic results: AMG 108 serum concentrations**. **(a) **part a, intravenous administration; **(b) **part a, subcutaneous administration; **(c) **part b, subcutaneous administration. IV, intravenous; LLOQ, lower limit of quantification; n*M*, nanomole; SC, subcutaneous.

In Part A (Figure 3a and 3b), mean AMG 108 serum concentration-time profiles generally exhibited nonlinear PK, and serum concentrations increased more than dose proportionately. After a 30-minute IV infusion (100 mg, 300 mg) on day 1, C_max _and AUC_0-τ _increased approximately dose proportionately (2.6-fold and 3.2-fold, respectively; Table 3). However, the C_max _and AUC_0-τ _values increased greater than dose proportionately for SC administration in Part A. After the first SC dose (75 mg, 300 mg) on day 1, C_max _increased 8.2-fold, and AUC_0-τ _increased 17.3-fold for a fourfold dose increase (Table 3). Because of the nonlinear nature of the PK data and insufficient data for the terminal phase of the concentration-time profile, the half-life of AMG 108 could not be determined.

**Table 3:** Pharmacokinetic data after intravenous and subcutaneous administration of AMG 108

	AMG 108 dose and route of administration			
	100 mg IV	300 mg IV	300 mg SC	75 mg SC
**Day 1 (first dose)**	(*n *= 12)	(*n *= 12)	(*n *= 12)	(*n *= 12)
**C_max _(n*M*; mean (SD))**	312 (72)	806 (112)	220 (58)	26.7 (12.9)
**T_max _(hr; median (range))**	1.0 (0.5-24.0)	1.0 (0.5-12.0)	144 (144-335)	144 (48-145)
**AUC_0-τ _(n*M*; mean (SD))**	2,580 (665)	8,280 (1,690)	4,230 (1,210)	244 (156)
**Trough concentration (n*M*; mean (SD))**	14.9 (15.2)	148 (48)	96.3 (38.3)	BQL
**Day 56 (third dose)**	(*n *= 12)	(*n *= 8)^a^	(*n *= 12)	(*n *= 6)^a^
**C_max _(n*M*; mean (SD))**	315 (91)	960 (192)	397 (123)	41.4 (11.1)
**T_max _(hr; median (range))**	0.51 (0.50-8.0)	0.51 (0.50-8.1)	168 (48-336)	48 (48-169)
**AUC_0-τ _(n*M*; mean (SD))**	2,700 (1,360)	12,000 (2,230)	8,610 (3,010)	449 (213)
**Trough concentration (n*M*, mean (SD))**	22.5 (32.3)	257 (93)	216 (100)	BQL

AUC_0-τ,_area under the concentration-time curve, estimated by using a linear/log trapezoidal method from days 1 to 28 for the first dose and days 56 to 84 for the third dose; BQL, below the quantification limit; C_max_, maximum observed serum concentration; hr, hour; IV, intravenous; n*M*, nanomolar; SC, subcutaneous; SD. standard deviation; T_max_, time to C_max_. ^a^Patients who did not receive all 3 doses of study medication were excluded from group mean for day 56.

Additionally, after a single SC dose of 300 mg AMG 108 in Part A, mean synovial fluid concentrations were 60.3 and 55.4 n*M *at days 7 and 14, respectively; before the day-28 dose, the concentration was 39.0 n*M*. Thus, monthly SC administration of 300 mg AMG 108 appeared to provide adequate drug exposure above the estimated IC_90 _value (approximately 13.5 n*M *(per *ex vivo *data not shown)) in both serum and synovial fluid.

In Part B (Figure 3c), serum concentrations observed after three SC administrations of 300 mg AMG 108 were generally within the range observed during Part A; however, the results should be interpreted with caution, given the small sample size in Part A (*n *= 12) compared with Part B (*n *= 60 to 65).

An analysis of mean synovial fluid to serum drug concentration ratios for nonlavage samples showed a range from approximately 2% to 45%; however, sample sizes in this analysis were extremely small (*n *= 1 to 3 in each group]. The ratios for the 75-mg and 300-mg SC groups were lower on day 2 (2.07% (*n *= 2) and 9.63% (*n *= 3), respectively) than the ratios for the 100-mg and 300-mg IV groups (23.5% (*n *= 2) and 26.2% (*n *= 3), respectively), suggesting that there might be a time delay between absorption of AMG 108 after SC injection and subsequent distribution to the synovial fluid.

### Clinical effects

**Figure 4:**
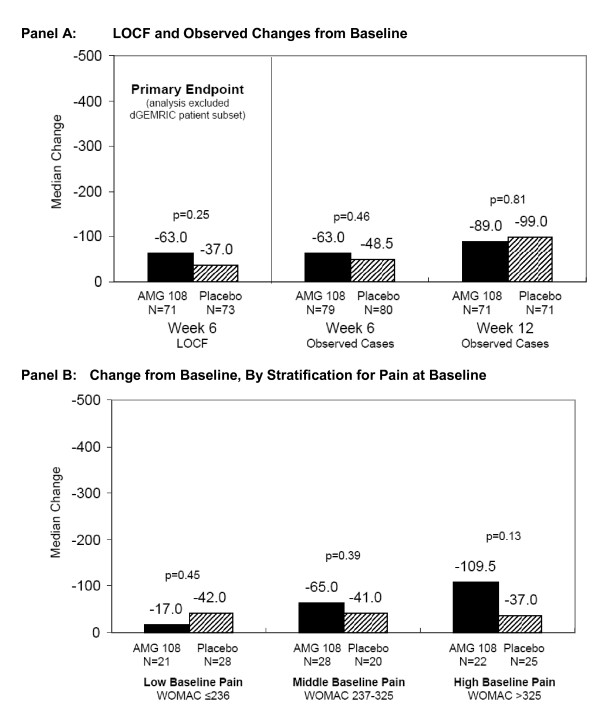
**Median change from baseline in WOMAC pain scores**. **(a) **Last observation carried forward (LOCF) and observed change from baseline. Note: Primary LOCF analysis at week 6 includes only the evaluable patients randomized to the WOMAC study (one AMG 108 patient was not evaluable); observed analyses at weeks 6 and 12 include all evaluable patients. **(b) **Change from baseline, by stratification for pain at baseline. dGEMRIC, delayed gadolinium-enhanced magnetic resonance imaging of cartilage; LOCF, last observation carried forward; WOMAC, Western Ontario and McMaster Universities osteoarthritis index pain score.

The mean WOMAC pain scores at baseline were similar in the AMG 108 group (278.8) and placebo group (268.4) in Part B. Both groups had decreased WOMAC pain scores from baseline at week 6, the primary efficacy end point. Although the difference was not statistically significant, patients in the AMG 108 group had numerically greater improvement in pain than did placebo patients (median LOCF change, -63.0 versus -37.0, respectively; *P *= 0.25; Figure 4a). Rescue therapy with NSAIDs or analgesics was required for six of 72 patients in the AMG 108 group and 10 of 73 patients in the placebo group during the study; however, analyses excluding these patients or imputing their WOMAC pain scores after rescue therapy (by using last observation before therapy) did not affect the overall results.

When patients were stratified for pain at baseline, a trend in improvement from baseline to week 6 was observed in the AMG 108 group versus the placebo group (Figure 4B). When analyzed from lowest to highest baseline pain tertile, differences in pain score change from baseline increased from -17.0 to -65.0 to -109.5 with AMG 108, whereas with placebo, it remained at approximately -40.0 for each tertile; however, the differences were not statistically significant for any tertile (*P *= 0.45, 0.39, and 0.13, respectively).

The placebo group showed greater, but statistically insignificant, median change from baseline at week 6 than did the AMG 108 group in the other two WOMAC index domains of physical function score (-170 versus -155, respectively; *P *= 0.95) and stiffness (-24 versus -21, respectively; *P *= 0.59). Composite WOMAC scores at week 6 showed approximately equal changes in both groups (-270 versus -263, placebo versus AMG 108, respectively; *P *= 0.97). By week 12 of the study, patients in the AMG 108 group had greater median improvements in the WOMAC composite score (pain, physical function, and stiffness), compared with the placebo group (median observed change in composite score -436 versus -314, respectively), although the difference was not statistically significantly different (*P *= 0.48). Changes in additional end points (for example, physician and patient global assessments of pain, the SF-36, and the EQ-5D) were insignificant and are not reported herein.

The study was suspended by the sponsor to investigate the fatal serious adverse event in a patient in the AMG 108 300-mg IV group in Part A, after which the study was resumed and completed. The AMG 108 program in OA was terminated because of lack of demonstrable clinical benefit.

## Discussion

In this two-part, randomized, double-blind multiple-dose study in patients with OA of the knee, AMG 108 was well tolerated when administered SC (75 and 300 mg) or IV (100 and 300 mg). Most AEs, infectious AEs, serious AEs and infections, and withdrawals from study due to AEs occurred at similar rates in the AMG 108 and placebo groups. One death was reported in an 80-year-old patient (Part A, 300-mg IV AMG 108; complications of lobar pneumonia). AMG 108 serum concentration-time profiles generally exhibited nonlinear PK during the study, and levels of study drug in both serum and synovial fluid appeared to be adequate for IL-1 inhibition. A nonstatistically significant trend toward clinical effect was observed, particularly in the subset with high VAS pain scores at baseline; the AMG 108 group in Part B had numerically greater but statistically insignificant improvement in pain than did the placebo group, as shown by the WOMAC scores (median change, -63.0 versus -37.0, respectively). However, the clinical relevance of the changes is not clear. The expected effects of IL-1 inhibition were demonstrated in decreased CRP and mean neutrophil counts.

It has been shown that IL-1 plays an important role in preclinical models of OA [13] and in studies of human cartilage and bone [18]. IL-1 may also have an effect on the pain of OA, although this was not observed in a study by Richette *et al. *[7] However, despite a significant difference between the AMG 108 and placebo groups in CRP values, a biologic effect of IL-1 inhibition, the primary end point (significant improvement in WOMAC-measured pain) was not reached in this study, nor was it reached in a study of autologous IL-1ra (orthokin) by Yang *et al. *(16.8% versus 16.5%, orthokin versus placebo, respectively) [19].

Decreases in neutrophil counts have been observed in RA patients receiving anti-IL-1 therapies; results of clinical studies in OA patients have also shown these changes [13,16,20]. In the current study, AMG 108 administration was associated with decreases in ANCs and platelets, which returned to baseline values at the end of study. Infections appeared to occur at greater frequencies in the high-dose SC groups compared with placebo; however, the incidence of serious infections was similar in the AMG 108 and placebo groups.

Pharmacokinetic evaluation of both synovial fluid and serum in this study suggested that adequate IL-1 inhibition should have been achieved with the dose and frequency of AMG 108 administered (trough concentrations exceeded the IC_90 _value for IL-1β-induced IL-6 inhibition at trough levels in both serum and synovial fluid). However, it was not possible to evaluate the penetration of AMG 108 into the deeper cartilage layers in this study, and the availability of the drug to chondrocytes in cartilage lacunae remains a possible limitation of this strategy for IL-1 inhibition. Another possible limitation was that patients were required to have only one knee evaluated, and it was not required to be erosive; eligibility criteria that required more inflammatory or widespread OA might have resulted in a more robust clinical effect. Although a nonsignificant trend toward clinical benefit was suggested in this study in subjects with a high baseline VAS, the magnitude of the benefit was not considered large enough to pursue development of AMG 108 for the OA indication.

## Conclusions

Overall, the safety profile of AMG 108 was similar to placebo in this short, 3-month study, but minimal if any clinical benefit was observed in patients with OA of the knee. Potential areas for further research of IL-1 inhibition in OA may include varieties of OA that respond poorly to available therapies (for example, inflammatory OA or erosive OA of the hands).

## Abbreviations

AE: adverse event; ANC: absolute neutrophil count; BMI: body mass index; CRP: C-reactive protein; dGEMRIC: delayed gadolinium-enhanced magnetic resonance imaging of cartilage; GAG: glycosaminoglycan; IA: intraarticular; IL-1R1: interleukin-1 receptor type 1; IV: intravenous; LOCF: last observation carried forward; MRI: magnetic resonance imaging; NSAID: nonsteroidal anti-inflammatory drug; OA: osteoarthritis; PK: pharmacokinetic; SAE: serious adverse event; SC: subcutaneous; WOMAC: Western Ontario and McMaster Universities osteoarthritis index.

## Competing interests

At the time the study was performed, SB Cohen was a consultant for Amgen Inc, Genentech, Biogen-IDEC, Merck, Sanofi-Aventis, Proctor and Gamble, Pfizer, Centocor, Scios, Bristol Meyers Squibb, and Wyeth-Ayerst; S Proudman was a consultant for Amgen Inc, Actelion, Pfizer, and Glaxo-Smith-Klein; A Kivitz, F Burch, and D Burstein were consultants for Amgen Inc; and J Donohue was a consultant for Amgen Inc, Genentech, and Bristol Myers Squibb. Y-N Sun, C Banfield, MS Vincent, L Ni, and DJ Zack are employees of Amgen Inc.

## Authors' contributions

Y-N Sun, C Banfield, MS Vincent, L Ni, and DJ Zack made substantial contributions to the study concept or design. SB Cohen, S Proudman, AJ Kivitz, FX Burch, JP Donohue, and D Burstein assisted with the acquisition of the data. Y-N Sun and L Ni performed data analysis. All authors assisted with interpretation of the data, helped to draft and revise the manuscript for intellectual content, and approved the final manuscript before submission to the journal.

## Supplementary Material

Additional file 1 **dGEMRIC imaging, analysis and results**. Click here for file

## References

[B1] Cooper C Klippel JH, Dieppe PA Osteoarthritis and related disorders: epidemiology Rheumatology 1998 2 London: Mosby 8.2.1-8

[B2] Samuels J Krasnokutsky S Abramson SB Osteoarthritis: a tale of three tissues Bull NYU Hosp Jt Dis 2008 66 244 250 18937640

[B3] LeGrand A Fermor B Fink C Pisetsky DS Weinberg JB Vail TP Guilak F Interleukin-1, tumor necrosis factor alpha, and interleukin-17 synergistically up-regulate nitric oxide and prostaglandin E2 production in explants of human osteoarthritic knee menisci Arthritis Rheum 2001 44 2078 2083 10.1002/1529-0131(200109)44:9<2078::AID-ART358>3.0.CO;2-J 11592370

[B4] Murakami S Lefebvre V de Crombrugghe B Potent inhibition of the master chondrogenic factor Sox9 gene by interleukin-1 and tumor necrosis factor-alpha J Biol Chem 2000 275 3687 3692 10.1074/jbc.275.5.3687 10652367

[B5] Petrov R MacDonald MH Tesch AM Benton HP Inhibition of adenosine kinase attenuates interleukin-1- and lipopolysaccharide-induced alterations in articular cartilage metabolism Osteoarthritis Cartilage 2005 13 250 257 10.1016/j.joca.2004.12.004 15727892

[B6] Presle N Cipolletta C Jouzeau JY Abid A Netter P Terlain B Cartilage protection by nitric oxide synthase inhibitors after intraarticular injection of interleukin-1beta in rats Arthritis Rheum 1999 42 2094 2102 10.1002/1529-0131(199910)42:10<2094::AID-ANR9>3.0.CO;2-Y 10524680

[B7] Richette P Francois M Vicaut E Fitting C Bardin T Corvol M Savouret JF Rannou F A high interleukin 1 receptor antagonist/IL-1beta ratio occurs naturally in knee osteoarthritis J Rheumatol 2008 35 1650 1654 18597398

[B8] Vuolteenaho K Moilanen T Hamalainen M Moilanen E Regulation of nitric oxide production in osteoarthritic and rheumatoid cartilage: role of endogenous IL-1 inhibitors Scand J Rheumatol 2003 32 19 24 10.1080/03009740310000355 12635941

[B9] Caron JP Fernandes JC Martel-Pelletier J Tardif G Mineau F Geng C Pelletier JP Chondroprotective effect of intraarticular injections of interleukin-1 receptor antagonist in experimental osteoarthritis: suppression of collagenase-1 expression Arthritis Rheum 1996 39 1535 1544 10.1002/art.1780390914 8814066

[B10] Pelletier JP Caron JP Evans C Robbins PD Georgescu HI Jovanovic D Fernandes JC Martel-Pelletier J In vivo suppression of early experimental osteoarthritis by interleukin-1 receptor antagonist using gene therapy Arthritis Rheum 1997 40 1012 1019 10.1002/art.1780400604 9182910

[B11] Fernandes J Tardif G Martel-Pelletier J Lascau-Coman V Dupuis M Moldovan F Sheppard M Krishnan BR Pelletier JP In vivo transfer of interleukin-1 receptor antagonist gene in osteoarthritic rabbit knee joints: prevention of osteoarthritis progression Am J Pathol 1999 154 1159 1169 10.1016/S0002-9440(10)65368-0 10233854 PMC1866546

[B12] Frisbie DD Ghivizzani SC Robbins PD Evans CH McIlwraith CW Treatment of experimental equine osteoarthritis by in vivo delivery of the equine interleukin-1 receptor antagonist gene Gene Ther 2002 9 12 20 10.1038/sj.gt.3301608 11850718

[B13] Fernandes JC Martel-Pelletier J Pelletier JP The role of cytokines in osteoarthritis pathophysiology Biorheology 2002 39 237 246 12082286

[B14] Bashir A Gray ML Hartke J Burstein D Nondestructive imaging of human cartilage glycosaminoglycan concentration by MRI Magn Reson Med 1999 41 857 865 10.1002/(SICI)1522-2594(199905)41:5<857::AID-MRM1>3.0.CO;2-E 10332865

[B15] Ren K Torres R Role of interleukin-1beta during pain and inflammation Brain Res Rev 2009 60 57 64 10.1016/j.brainresrev.2008.12.020 19166877 PMC3076185

[B16] Chevalier X Giraudeau B Conrozier T Marliere J Kiefer P Goupille P Safety study of intraarticular injection of interleukin 1 receptor antagonist in patients with painful knee osteoarthritis: a multicenter study J Rheumatol 2005 32 1317 1323 15996071

[B17] Altman R Asch E Bloch D Bole G Borenstein D Brandt K Christy W Cooke TD Greenwald R Hochberg M Development of criteria for the classification and reporting of osteoarthritis: classification of osteoarthritis of the knee: Diagnostic and Therapeutic Criteria Committee of the American Rheumatism Association Arthritis Rheum 1986 29 1039 1049 10.1002/art.1780290816 3741515

[B18] Pelletier JP DiBattista JA Roughley P McCollum R Martel-Pelletier J Cytokines and inflammation in cartilage degradation Rheum Dis Clin North Am 1993 19 545 568 8210574

[B19] Yang KG Raijmakers NJ van Arkel ER Caron JJ Rijk PC Willems WJ Zijl JA Verbout AJ Dhert WJ Saris DB Autologous interleukin-1 receptor antagonist improves function and symptoms in osteoarthritis when compared to placebo in a prospective randomized controlled trial Osteoarthritis Cartilage 2008 16 498 505 10.1016/j.joca.2007.07.008 17825587

[B20] Chevalier X Goupille P Beaulieu AD Burch FX Bensen WG Conrozier T Loeuille D Kivitz AJ Silver D Appleton BE Intraarticular injection of anakinra in osteoarthritis of the knee: a multicenter, randomized, double-blind, placebo-controlled study Arthritis Rheum 2009 61 344 352 10.1002/art.24096 19248129

