# Supplementary material for: A randomized, double-blind study of AMG 108 (a fully human monoclonal antibody to IL-1R1) in patients with osteoarthritis of the knee
Source: Arthritis Res Ther. 2011 Jul 29;13(4):R125. doi: 10.1186/ar3430 (PMC3239365; doi:10.1186/ar3430)
Supplement: Additional file 1 — dGEMRIC imaging, analysis and results. [file ar3430-S1.DOC]

**Additional File 1: dGEMRIC Imaging, Analysis, and Results**

**in**

**Cohen et al: A randomized, double-blind study of AMG 108 (a fully human monoclonal antibody to IL‑1R1) in patients with osteoarthritis of the knee**

**dGEMRIC Imaging and Analysis**

Cartilage integrity was assessed via delayed gadolinium-enhanced magnetic resonance imaging (MRI) of cartilage (dGEMRIC) in a small substudy of 15 patients in Part B; images were taken at baseline and at weeks 6 and 20.

Patients were excluded from the dGEMRIC substudy if they had a known or suspected reaction to gadolinium, an implanted metallic device or prosthetic, or any other contraindication to MRI. dGEMRIC involves injection of an MRI hydrophilic contrast agent Gd(DTPA)^2-^[21, 22] with subsequent imaging after a delay of 90 minutes to enable the contrast agent to penetrate the cartilage tissue; because the contrast agent is negatively charged, it will distribute in higher concentration in areas of cartilage with lower (negatively charged, i.e. degraded) glycosaminoglycan (GAG) concentration and will be relatively low in normal (GAG-abundant) cartilage. The studies were performed using a 3T short-bore GE Signa MRI system and a quadknee coil. Sequence parameters for each sequence were predetermined. The dGEMRIC Index refers to the longitudinal relaxation time after penetration of Gd(DTPA)^2-^ [23] and is reported as a T1 value (range 50–1500 msec, with lower scores indicating more disease).[22] Since T1 is higher with lower contrast agent concentration (higher GAG; e.g. T1 value above 500 msec), a positive change in this index is thought to represent an increase in the content of GAGs in cartilage, thus an improvement in OA disease status.[21, 23, 24]

The dGEMRIC substudy was an exploratory endpoint, and no inferential statistical analysis was performed.

**dGEMRIC Results**

Fifteen patients (AMG 108 n=8; placebo n=7) enrolled in the dGEMRIC substudy underwent MRI evaluation with contrast at week 6 and end-of-study to assess cartilage components of the index knee pre- and post-treatment. The mean change from baseline in dGEMRIC index in the medial femoral condyle was positive for the AMG 108 group at week 6 (T1 value 20.96 msec, SD 41.66), compared with the placebo group (T1 value -2.67 msec, SD 78.74). A positive change in dGEMRIC index is thought to represent an increase in the content of GAGs in cartilage,[25, 26] e.g, a dGEMRIC index score above 500 msec is considered high, 400‑500 msec is considered mid-range, and below 400 msec is considered low.[23]

**Table 1** shows the baseline, week 6, and week 20 medial femoral condyle T1 values for each of the 15 patients in the dGEMRIC substudy. **Figure 1** shows example images from the 2 patients in the study. Higher dGEMRIC Indices reflect improved cartilage status; therefore, the changes from the red end of the scale to yellow-green indicate improved cartilage composition and vice-versa. These changes are most apparent in the medial femoral cartilage.

**Table 1, Baseline, week 6, and week 20 medial femoral condyle T1 values for each of the 15 patients in the dGEMRIC substudy.**

| **dGEMRIC Index at Medial Femoral Condyle (T1 Value; msec)** | | | | |
| --- | --- | --- | --- | --- |
| **Patient No.** | **Treatment** | **Baseline** | **Week 6** | **Week 20** |
| 5001 | AMG 108 (300 mg SC) | 620.3 | 615 | 568.7 |
| 5008 | AMG 108 (300 mg SC) | 455.7 | 457 | 463 |
| 5014 | AMG 108 (300 mg SC) | 615 | 626.7 | 633.7 |
| 5015 | AMG 108 (300 mg SC) | 437.3 | 553.7 | 428 |
| 5016 | AMG 108 (300 mg SC) | 578.7 | 563 | 600.7 |
| 5018 | AMG 108 (300 mg SC) | 643.7 | 647.3 | 646.7 |
| 5020 | AMG 108 (300 mg SC) | 627.7 | 664.3 | 608.3 |
| 5023 | AMG 108 (300 mg SC) | 460.7 | 479.7 | 455.3 |
| 5002 | Placebo | 497.3 | 396.7 | Not available |
| 5004 | Placebo | 491.3 | 604 | 447 |
| 5005 | Placebo | 564.3 | Not available | Not available |
| 5009 | Placebo | 704 | 692.7 | 559.3 |
| 5010 | Placebo | 597.7 | 633.3 | 603 |
| 5021 | Placebo | 544.3 | 466 | 530.7 |
| 5024 | Placebo | 707 | 733 | Not available |
| The dGEMRIC Index refers to the longitudinal relaxation time after penetration of Gd(DTPA)^2-^ and is reported as a T1 value (range 50–1500 msec, with lower scores indicating more disease). A dGEMRIC index score above 500 msec is considered high, 400‑500 msec is considered mid-range, and below 400 msec is considered low.[22, 23] | | | | |

**Figure 1. Examples of dGEMRIC Index at the Medial Femoral Condyle in Two Patients at Baseline, Week 6, and Week 20.** Higher dGEMRIC Indices reflect improved cartilage status; therefore, the changes from the red end of the scale to yellow-green indicate improved cartilage composition and vice-versa. These changes are most apparent in the medial femoral cartilage*.*
